# Crystal structure and Hirshfeld surface analysis of 2-azido-*N*-(4-fluoro­phen­yl)acetamide

**DOI:** 10.1107/S2056989022006764

**Published:** 2022-07-29

**Authors:** Mohcine Missioui, Walid Guerrab, Abdulsalam Alsubari, Joel T. Mague, Youssef Ramli

**Affiliations:** a Laboratory of Medicinal Chemistry, Drug Sciences Research Center, Faculty of Medicine and Pharmacy, Mohammed V University in Rabat, Morocco; b Laboratory of Medicinal Chemistry, Faculty of Clinical Pharmacy, 21 September University, Yemen; c Department of Chemistry, Tulane University, New Orleans, LA 70118, USA; Katholieke Universiteit Leuven, Belgium

**Keywords:** crystal structure, azide, acetamide, hydrogen bond, Hirshfeld surface

## Abstract

The asymmetric unit of the title compound, C_8_H_7_FN_4_O, consists of two independent mol­ecules differing in the orientation of the azido group. Each mol­ecule forms N—H⋯O hydrogen-bonded chains along along the *c*-axis direction with its symmetry-related counterparts and the chains are connected by C—F⋯π(ring), C=O⋯π(ring) and slipped π-stacking inter­actions. A Hirshfeld surface analysis of these inter­actions was performed.

## Abstract 2

The asymmetric unit consists of two independent mol­ecules differing in the orientation of the azido group. Each mol­ecule forms N—H⋯O hydrogen-bonded chains extending along the *c*-axis direction with its symmetry-related counterparts. The chains are connected by C—F⋯π(ring), C=O⋯π(ring) and slipped π-stacking inter­actions. A Hirshfeld surface analysis of these inter­actions was performed.

## 1. Chemical context

Azides are a class of versatile organic compounds having the basic structure *R*N_3_ where *R* can be an alkyl, acyl or aryl group. They have found valuable applications in medicinal chemistry (Contin *et al.*, 2019 ▸) and mol­ecular biology (Ahmed & Abdallah, 2019 ▸). On the other hand, amide bonds are a key structural unit in many physiologically active compounds and have ubiquitous presence in biopolymers such as proteins and glycoproteins (Cheng *et al.*, 2016 ▸; Pattabiraman & Bode, 2011 ▸; Zheng *et al.*, 2016 ▸). Acetamides are useful building blocks for the preparation of biologically active natural products, especially depsipeptide compounds. In particular, *N*-aryl­acet­amides are significant inter­mediates for the synthesis of medicinal, agrochemical, and pharmaceutical compounds (Valeur & Bradley, 2009 ▸; Allen & Williams, 2011 ▸; Missioui *et al.*, 2021 ▸; Missioui *et al.*, 2022*a* ▸,*b* ▸). They have been identified as inhibitors of me­thio­nine amino­peptidase-2 and HIV protease, display potent anti­tumor activity, and play an important role in medicinal chemistry. As a result of the significance of this core, and in a continuation of our research efforts to synthesize *N*-aryl­acetamide-based heterocycles (Missioui *et al.*, 2020 ▸; Al-Taifi *et al.*, 2021 ▸; Guerrab *et al.*, 2021 ▸; Missioui *et al.*, 2022*c* ▸,*d* ▸), we report here the synthesis, mol­ecular and crystal structures and a Hirshfeld surface analysis of the title compound. 
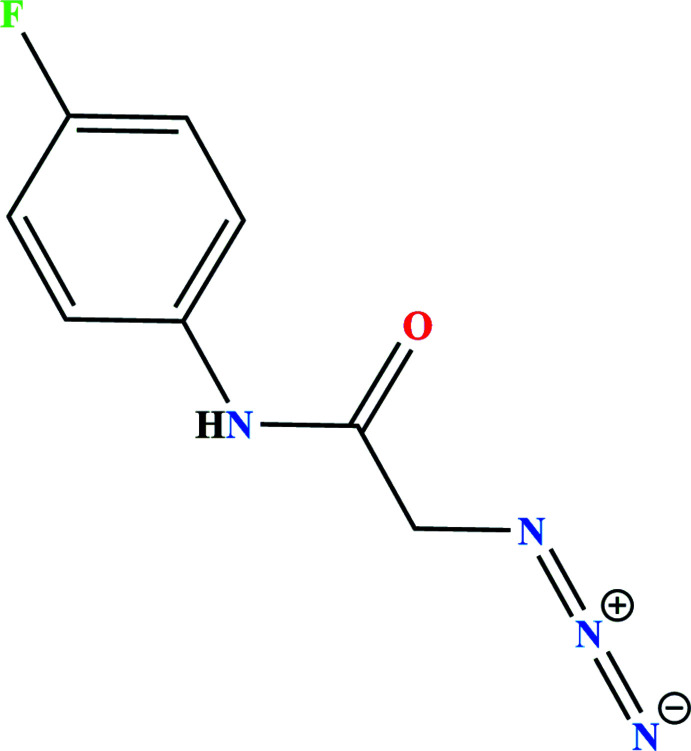


## 2. Structural commentary

**Figure 1:**
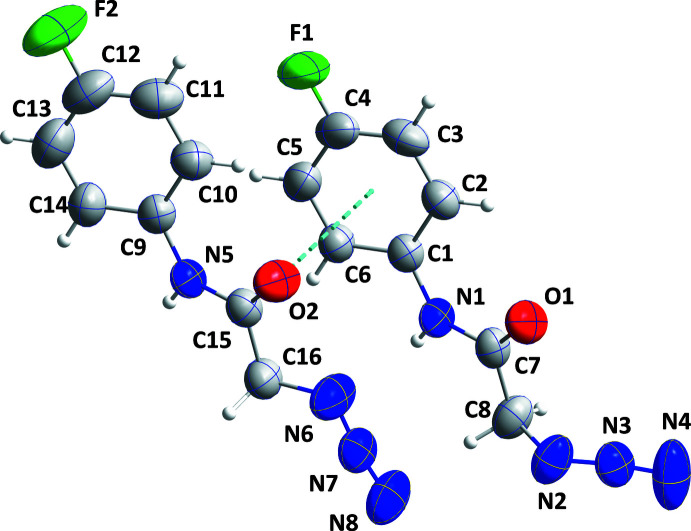
The asymmetric unit with labeling scheme and 50% probability ellipsoids. The C15=O2⋯*Cg*1 inter­action is depicted by a dashed line.

**Figure 2:**
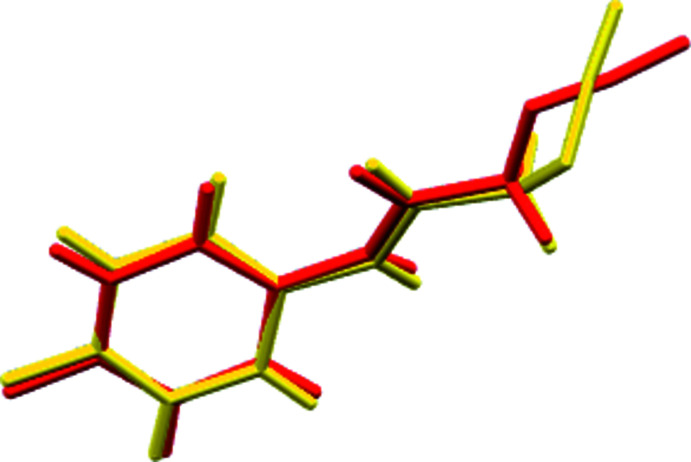
Overlay of the two mol­ecules in the asymmetric unit. The yellow mol­ecule contains O1 while the red one contains O2.

The asymmetric unit consists of two independent mol­ecules differing moderately in conformation and connected by a weak C15=O2⋯*Cg*1 inter­action [*Cg*1 is the centroid of the C1–C6 benzene ring; O2⋯*Cg*1 = 3.904 (2) Å, C15⋯*Cg*1 = 3.902 (2) Å, C15=O2⋯*Cg*1 = 80.88 (13)°] as shown in Fig. 1 ▸. The conformational difference is primarily in the orientation of the azide groups (Fig. 2 ▸). Thus the N3—N2—C8–C7 torsion angle between the planes defined by N1/C7/C8/O1 and C8/N2/N3/N4 is −106.1 (2)° while the corresponding dihedral angle in the other mol­ecule is −175.4 (2)°. The dihedral angle between the plane defined by N1/C7/C8/O1 and that of the C1-C6 ring is 21.85 (13)° while the corresponding angle (N7—N6—C16—C15) in the other mol­ecule is the same within experimental error. By comparison, in the *p*-tolyl analog (Missioui *et al.*, 2022*e* ▸), which has three independent mol­ecules in the asymmetric unit, the dihedral angles between the N/C/C/O and C/N/N/N planes are 7.6 (2), 86.34 (19) and 7.03 (19)° while those between the N/C/C/O and phenyl ring planes are 24.2 (2), 22.58 (10) and 15.38 (10)°.

## 3. Supra­molecular features

**Figure 3:**
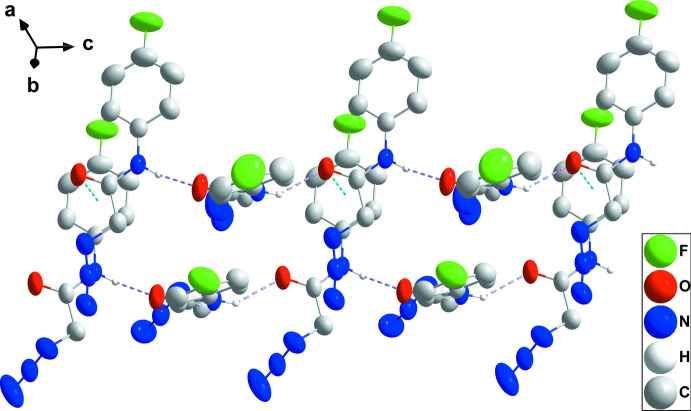
Perspective view of the chain structure with N—H⋯O hydrogen bonds and C15=O2⋯*Cg*1 inter­actions depicted, respectively, by violet and light-blue dashed lines. Non-inter­acting hydrogen atoms are omitted for clarity.

**Figure 4:**
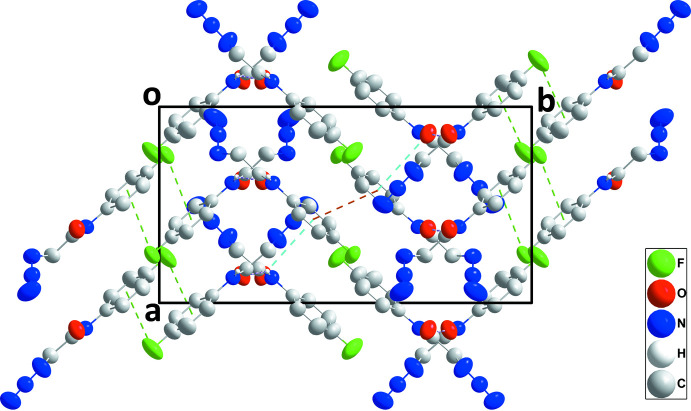
Packing viewed along the *c*-axis direction showing the linking of chains *via* C—F⋯π(ring) (green dashed lines) and C15=O2⋯*Cg*1 (light-blue dashed lines) and slipped π-stacking (orange dashed lines) inter­actions. N—H⋯O hydrogen bonds and non-inter­acting hydrogen atoms are omitted for clarity.

In the crystal, the mol­ecule containing O1 is linked into chains extending along the *c*-axis direction by N1—H1⋯O1^i^ hydrogen bonds [symmetry code: (i) *x*, −*y* + 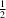 , *z* + 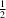 ], while N5—H5*A*⋯O2^i^ hydrogen bonds form parallel chains for the second independent mol­ecule (Table 1 ▸ and Fig. 3 ▸). The chains are linked by C4—F1⋯*Cg*1^ii^ and C12—F2⋯*Cg*2^iii^ [*Cg*2 is the centroid of the C9–C14 benzene ring; symmetry codes: (ii) −*x* + 1, −*y* + 1, −*z* + 1; (iii) −*x* + 2, −*y* + 1, −*z* + 2] inter­actions as well as by the C15=O2⋯*Cg*1 inter­action noted above and weak, slipped π-stacking between centrosymmetrically related C1–C6 benzene rings [centroid–centroid = 3.8661 (13) Å, slippage = 1.6 Å] (Table 1 ▸ and Fig. 4 ▸). For the related *p*-tolyl analog (Missioui *et al.*, 2022*e* ▸) each independent mol­ecule forms chains with its symmetry-related counterparts through N—H⋯O hydrogen bonds. There do not appear to be significant inter­molecular inter­actions between the chains although it is possible that very weak C=O⋯π(ring) inter­actions exist.

## 4. Database survey

A search of the Cambridge Structural Database (CSD, version 5.43, updated to March 2022; Groom *et al.*, 2016 ▸) with the search fragment **A** gave eleven hits of which three contained the 2-azido­acetamide unit while 30 hits resulted from a search with fragment **B**, of which six contained the 2-azido­acetamide unit. 
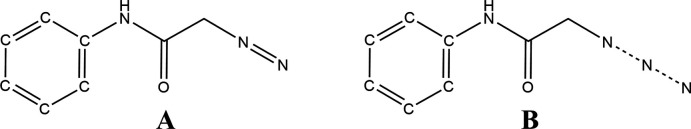


In the first group, the aromatic ring has a –CO_2_Et group in the 2-position (ARAPIU: Yassine *et al.*, 2016*a* ▸), the second has ^*i*^PrS– groups in the 2- and 3-positions (CEMRUJ: Okamura *et al.*, 2013 ▸) and the last has a –CO_2_^*n*^Bu group in the 2-position (OVIBAY: Yassine *et al.*, 2016*b* ▸). The six relevant structures in the second group include ones with an unsubstituted phenyl group (ASEDIO: Guerrab *et al.*, 2021 ▸) and those with the 4-position containing –NO_2_ (QAGNOF: Missioui *et al.*, 2020 ▸), HC≡C– (DAPYOM: Madhusudhanan *et al.*, 2021 ▸), MeO– (TARHIH: Missioui *et al.*, 2021 ▸) and 2-acet­oxy­methyl-3,4,5-triacet­oxy-tetra­hydro-2*H*-pyran-6-yl-O– (BEBPIJ: Cecioni *et al.*, 2012 ▸). The sixth has Cl at the 4-position and a 2-chloro­benzoyl substituent in the 2-position (VIFVOX: Cortes Eduardo *et al.*, 2012 ▸). In ARAPIU and OVIBAY, the amide hydrogens form intra­molecular N—H⋯O hydrogen bonds with the carboxyl oxygen while in CEMRUJ an intra­molecular inter­action of the amide hydrogen with the sulfur atom in the 2-position is postulated. Thus, none of these structures show the formation of chains as seen in the present case nor do any have more than one mol­ecule in the asymmetric unit. Among the others, ASEDIO has two independent mol­ecules in the asymmetric unit and it also, like QAGNOF and BEBPIJ, forms chains through N—H⋯O hydrogen bonds. In ASEDIO, the chains are connected by π-inter­actions between the terminal two nitro­gens of the azide group and a phenyl ring, while in QAGNOF the chains are connected by C—H⋯O and C—H⋯N hydrogen bonds. The remaining structures in the first group all contain the —N=N—C fragment while the remainder of the second group all contain triazoles as the N_3_-containing fragment and are not considered relevant to the present structure.

## 5. Hirshfeld surface analysis

**Figure 5:**
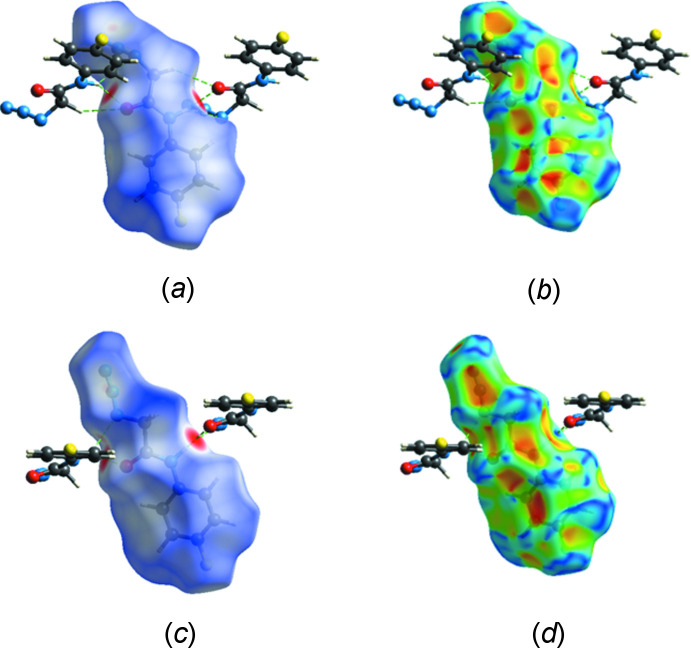
The (*a*) *d*_norm_ and (*b*) shape-index surfaces for the mol­ecule containing O1, and the (*c*) *d*_norm_ and (*d*) shape-index surfaces for the mol­ecule containing O2 together with the two closest mol­ecules of each type.

**Figure 6:**
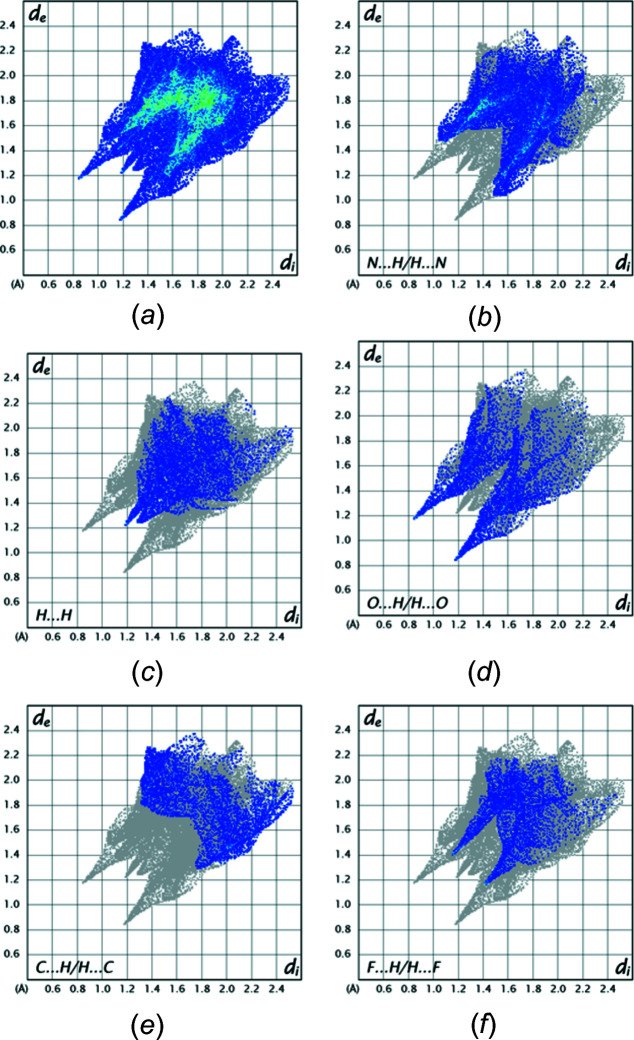
Fingerprint plots for the mol­ecule containing O1 showing inter­molecular inter­actions. (*a*) all; (*b*) N⋯H/H⋯N; (*c*) H⋯H; (*d*) O⋯H/H⋯O; (*e*) C⋯H/H⋯C; (*f*) F⋯H/H⋯F.

**Figure 7:**
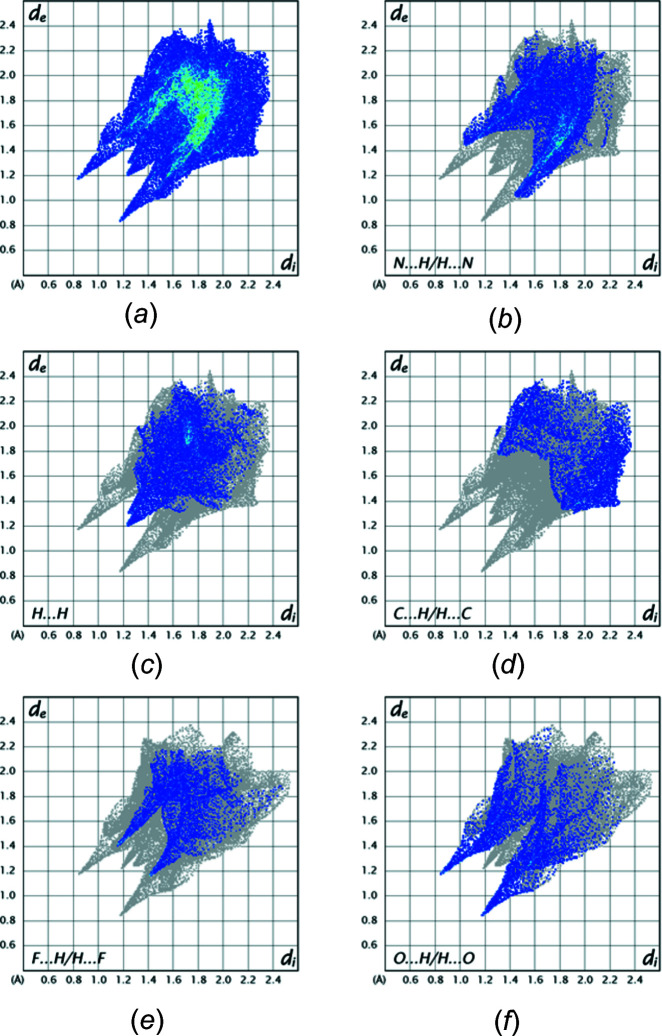
Fingerprint plots for the mol­ecule containing O2 showing inter­molecular inter­actions. (*a*) all; (*b*) N⋯H/H⋯N; (*c*) H⋯H; (*d*) C⋯H/H⋯C; (*e*) F⋯H/H⋯F; (*f*) O⋯H/H⋯O.

A Hirshfeld surface analysis was performed with *CrystalExplorer 21.5* (Spackman *et al.*, 2021 ▸) with the details of the pictorial output described in a recent publication (Tan *et al.*, 2019 ▸). Fig. 5 ▸ *a* and 5*c*, respectively, show the *d*_norm_ surfaces of the mol­ecule containing O1 and that containing O2 plotted over the range −0.4316 to 1.3253 in arbitrary units while Fig. 5 ▸ *b* and 5*d* show the corresponding shape-index functions. In both, two adjacent mol­ecules that are part of the hydrogen-bonded chains are included with the N—H⋯O and C—H⋯O inter­actions shown by dashed lines. The pattern of orange and blue triangles indicative of a π-inter­action is clearly evident in the lower part of Fig. 5 ▸ *b* and corresponds to the C4—F1⋯*Cg*1 inter­action. This is less clear in Fig. 5 ▸ *d* but the data in Table 1 ▸ clearly support a similar inter­action for this mol­ecule. Fig. 6 ▸ presents fingerprint plots for the mol­ecule containing O1 with Fig. 6 ▸ *a* showing all inter­molecular inter­actions and Fig. 6 ▸ *b*–6*f* those delineated into N⋯H/H⋯N (34.3%), H⋯H (13.5%), O⋯H/H⋯O (12.2%), C⋯H/H⋯C (11.9%) and F⋯H/H⋯F (9.7%), respectively. The two spikes in Fig. 6 ▸ *d* primarily represent the N—H⋯O hydrogen bonds but their breadth at longer values of *d*_i_ + *d*_e_ than at the tips indicate the contributions from C—H⋯O hydrogen bonds. Fig. 7 ▸ shows the fingerprint plots for the mol­ecule containing O2 with Fig. 7 ▸ *a* showing all inter­molecular inter­actions and Fig. 7 ▸ *b*–7*f* those delineated into N⋯H/H⋯N (28.8%), H⋯H (18.2%), C⋯H/H⋯C (12.6%), F⋯H/H⋯F (12.6%) and O⋯H/H⋯O (11.6%), respectively. Although the ordering of inter­actions based on their percentage of the total is not the same as in the other mol­ecule, the percentages are not greatly different between the two and the corresponding plots are very similar type by type.

## 6. Synthesis and crystallization

2-Chloro-*N*-(4-fluoro­phen­yl)acetamide (0.011 mol), and sodium azide (0.015 mol) were dissolved in a mixture of ethanol/water (70:30) and refluxed for 24 h at 353 K. After completion of the reaction (monitored by thin-layer chromatography, TLC), the 2-azido-*N*-(4-fluoro­phen­yl)acetamide that precipitated was filtered off and washed with cold water. A portion of the product was dissolved in hot ethanol, the solution was filtered, and the filtrate was left undisturbed for 7 days to form colorless, thick plate-like crystals.

Yield 69%, m.p. 358–360K, FT–IR (ATR, υ, cm^−1^) 3254 υ (N—H amide), 1027 υ (N—C amide), 1660 υ (C=O amide), 3073 υ(C—H_arom_), 1175 υ(C—N), 2961 υ(C—H, CH_2_), 2109 υ (N_3_), ^1^H NMR (DMSO–*d*_6_) δ ppm: 4.02 (2H, *s*, CH_2_), 6.93–7.11 (4H, *m*, *J* = 1.3 Hz, H_arom_), 10.05 (1H, *s*, NH), ^13^C NMR (DMSO–*d*_6_) δ ppm: 51.18 (CH_2_), 131.47 (C_arom_—N), 113.90–120.86 (C_arom_); 165.71 (C=O); HRMS (ESI–MS) (*m*/*z*) calculated for C_8_H_7_FN_4_O 194.18; found 194.1165.

## 7. Refinement

Crystal data, data collection and structure refinement details are summarized in Table 2 ▸. H atoms attached to carbon were placed in calculated positions (C—H = 0.95–0.99 Å) while those attached to nitro­gen were placed in locations derived from a difference map and their parameters adjusted to give N—H = 0.91 Å. All were included as riding contributions with isotropic displacement parameters 1.2–1.5 times those of the attached atoms.

## Supplementary Material

Crystal structure: contains datablock(s) global, I. DOI: 10.1107/S2056989022006764/vm2266sup1.cif

Structure factors: contains datablock(s) I. DOI: 10.1107/S2056989022006764/vm2266Isup3.hkl

Supporting information file. DOI: 10.1107/S2056989022006764/vm2266Isup3.cml

CCDC reference: 2183049

Additional supporting information:  crystallographic information; 3D view; checkCIF report

## Figures and Tables

**Table 1:** Hydrogen-bond geometry (Å, °) *Cg*1 and *Cg*2 are the centroids of the C1–C6 and C9–C14 benzene rings, respectively.

*D*—H⋯*A*	*D*—H	H⋯*A*	*D*⋯*A*	*D*—H⋯*A*
N1—H1⋯O1^i^	0.86	2.17	2.921 (2)	146
N5—H5*A*⋯O2^i^	0.86	2.13	2.885 (2)	146
C4—F1⋯*Cg*1^ii^	1.35 (1)	3.76 (1)	3.563 (2)	72 (1)
C12—F2⋯*Cg*2^iii^	1.36 (1)	3.98 (1)	3.942 (2)	79 (1)

Symmetry codes: (i) 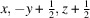 ; (ii) 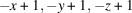 ; (iii) 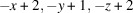 .

**Table 2:** Experimental details

Crystal data	
Chemical formula	C_8_H_7_FN_4_O
*M*_r_	194.18
Crystal system, space group	Monoclinic, *P*2_1_/*c*
Temperature (K)	296
*a*, *b*, *c* (Å)	10.8398 (7), 19.0207 (11), 9.3307 (5)
β (°)	112.378 (2)
*V* (Å^3^)	1778.93 (18)
*Z*	8
Radiation type	Cu *K*α
μ (mm^−1^)	1.00
Crystal size (mm)	0.47 × 0.25 × 0.15

Data collection	
Diffractometer	Bruker D8 VENTURE PHOTON 100 CMOS
Absorption correction	Multi-scan (*SADABS*; Krause *et al.*, 2015 ▸)
*T*_min_, *T*_max_	0.75, 0.87
No. of measured, independent and observed [*I* > 2σ(*I*)] reflections	12623, 3224, 2545
*R*_int_	0.034
(sin θ/λ)_max_ (Å^−1^)	0.603

Refinement	
*R*[*F* ^2^ > 2σ(*F* ^2^)], *wR*(*F* ^2^), *S*	0.053, 0.162, 1.06
No. of reflections	3224
No. of parameters	254
H-atom treatment	H-atom parameters constrained
Δρ_max_, Δρ_min_ (e Å^−3^)	0.45, −0.24

Computer programs: *APEX3* and *SAINT* (Bruker, 2016 ▸), *SHELXT/5* (Sheldrick, 2015*a* ▸), *SHELXL2018/3* (Sheldrick, 2015*b* ▸), *DIAMOND* (Brandenburg & Putz, 2012 ▸), and *SHELXTL* (Sheldrick, 2008 ▸).

## supplementary crystallographic information

### Crystal data

**Table:**

C_8_H_7_FN_4_O	*F*(000) = 800
*M**_r_* = 194.18	*D*_x_ = 1.450 Mg m^−^^3^
Monoclinic, *P*2_1_/*c*	Cu *K*α radiation, λ = 1.54178 Å
*a* = 10.8398 (7) Å	Cell parameters from 8481 reflections
*b* = 19.0207 (11) Å	θ = 2.3–68.3°
*c* = 9.3307 (5) Å	µ = 1.00 mm^−^^1^
β = 112.378 (2)°	*T* = 296 K
*V* = 1778.93 (18) Å^3^	Thick plate, colourless
*Z* = 8	0.47 × 0.25 × 0.15 mm

### Data collection

**Table:**

Bruker D8 VENTURE PHOTON 100 CMOS diffractometer	3224 independent reflections
Radiation source: INCOATEC IµS micro–focus source	2545 reflections with *I* > 2σ(*I*)
Mirror monochromator	*R*_int_ = 0.034
Detector resolution: 10.4167 pixels mm^-1^	θ_max_ = 68.5°, θ_min_ = 4.4°
ω scans	*h* = −12→11
Absorption correction: multi-scan (*SADABS*; Krause *et al.*, 2015)	*k* = −22→22
*T*_min_ = 0.75, *T*_max_ = 0.87	*l* = −11→11
12623 measured reflections	

### Refinement

**Table:**

Refinement on *F*^2^	Secondary atom site location: difference Fourier map
Least-squares matrix: full	Hydrogen site location: inferred from neighbouring sites
*R*[*F*^2^ > 2σ(*F*^2^)] = 0.053	H-atom parameters constrained
*wR*(*F*^2^) = 0.162	*w* = 1/[σ^2^(*F*_o_^2^) + (0.0828*P*)^2^ + 0.4718*P*] where *P* = (*F*_o_^2^ + 2*F*_c_^2^)/3
*S* = 1.06	(Δ/σ)_max_ < 0.001
3224 reflections	Δρ_max_ = 0.45 e Å^−^^3^
254 parameters	Δρ_min_ = −0.24 e Å^−^^3^
0 restraints	Extinction correction: *SHELXL 2018/3* (Sheldrick, 2015*b*), Fc^*^=kFc[1+0.001xFc^2^λ^3^/sin(2θ)]^-1/4^
Primary atom site location: dual	Extinction coefficient: 0.0033 (5)

### Special details

**Table:**

Geometry. All esds (except the esd in the dihedral angle between two l.s. planes) are estimated using the full covariance matrix. The cell esds are taken into account individually in the estimation of esds in distances, angles and torsion angles; correlations between esds in cell parameters are only used when they are defined by crystal symmetry. An approximate (isotropic) treatment of cell esds is used for estimating esds involving l.s. planes.
Refinement. Refinement of F^2^ against ALL reflections. The weighted R-factor wR and goodness of fit S are based on F^2^, conventional R-factors R are based on F, with F set to zero for negative F^2^. The threshold expression of F^2^ > 2sigma(F^2^) is used only for calculating R-factors(gt) etc. and is not relevant to the choice of reflections for refinement. R-factors based on F^2^ are statistically about twice as large as those based on F, and R- factors based on ALL data will be even larger. H-atoms attached to carbon were placed in calculated positions (C—H = 0.95 - 0.99 Å) while those attached to nitrogen were placed in locations derived from a difference map and their parameters adjusted to give N—H = 0.91 Å. All were included as riding contributions with isotropic displacement parameters 1.2 - 1.5 times those of the attached atoms.

### Fractional atomic coordinates and isotropic or equivalent isotropic displacement parameters (Å^2^)

**Table:**

	*x*	*y*	*z*	*U*_iso_*/*U*_eq_	
F1	0.75450 (16)	0.51934 (8)	0.5506 (2)	0.1053 (6)	
O1	0.37628 (16)	0.27865 (8)	0.11857 (16)	0.0704 (5)	
N1	0.38854 (15)	0.30447 (8)	0.36111 (17)	0.0528 (4)	
H1	0.357573	0.292390	0.429765	0.063*	
N2	0.23792 (19)	0.15768 (11)	0.1118 (2)	0.0780 (6)	
N3	0.1406 (2)	0.15796 (10)	−0.0097 (2)	0.0673 (5)	
N4	0.0587 (3)	0.15058 (17)	−0.1238 (3)	0.1192 (11)	
C1	0.48107 (18)	0.36020 (10)	0.4020 (2)	0.0494 (4)	
C2	0.5065 (2)	0.40201 (11)	0.2943 (2)	0.0614 (5)	
H2	0.461604	0.394037	0.188729	0.074*	
C3	0.5994 (2)	0.45573 (12)	0.3457 (3)	0.0725 (6)	
H3	0.617810	0.483972	0.274916	0.087*	
C4	0.6637 (2)	0.46685 (11)	0.5015 (3)	0.0702 (6)	
C5	0.6383 (2)	0.42787 (12)	0.6092 (3)	0.0675 (6)	
H5	0.681609	0.437309	0.714400	0.081*	
C6	0.54707 (19)	0.37419 (11)	0.5590 (2)	0.0569 (5)	
H6	0.529166	0.346752	0.631306	0.068*	
C7	0.34277 (18)	0.26790 (10)	0.2280 (2)	0.0517 (5)	
C8	0.2417 (2)	0.21221 (12)	0.2228 (3)	0.0655 (6)	
H8A	0.265262	0.191575	0.324847	0.079*	
H8B	0.154166	0.233482	0.193244	0.079*	
F2	1.2344 (2)	0.51818 (12)	0.9845 (3)	0.1438 (9)	
O2	0.86230 (17)	0.27797 (9)	0.55142 (18)	0.0760 (5)	
N5	0.87105 (16)	0.30220 (9)	0.79296 (17)	0.0546 (4)	
H5A	0.839332	0.289670	0.860681	0.065*	
N6	0.6704 (2)	0.18385 (13)	0.4918 (2)	0.0899 (7)	
N7	0.5847 (2)	0.14001 (11)	0.4654 (2)	0.0670 (5)	
N8	0.5019 (2)	0.10061 (15)	0.4215 (3)	0.0952 (8)	
C9	0.96341 (19)	0.35814 (10)	0.8354 (2)	0.0536 (5)	
C10	0.9881 (2)	0.40031 (12)	0.7283 (3)	0.0644 (5)	
H10	0.943298	0.392439	0.622692	0.077*	
C11	1.0801 (3)	0.45421 (14)	0.7798 (4)	0.0827 (7)	
H11	1.098105	0.482845	0.709361	0.099*	
C12	1.1445 (3)	0.46493 (15)	0.9364 (4)	0.0907 (8)	
C13	1.1195 (3)	0.42528 (16)	1.0435 (3)	0.0904 (8)	
H13	1.162906	0.434404	1.148769	0.108*	
C14	1.0292 (2)	0.37163 (13)	0.9929 (3)	0.0713 (6)	
H14	1.011492	0.343792	1.064795	0.086*	
C15	0.82697 (19)	0.26628 (10)	0.6592 (2)	0.0539 (5)	
C16	0.7313 (2)	0.20775 (12)	0.6530 (2)	0.0636 (5)	
H16A	0.778421	0.169287	0.719587	0.076*	
H16B	0.662987	0.224593	0.687969	0.076*	

### Atomic displacement parameters (Å^2^)

**Table:**

	*U*^11^	*U*^22^	*U*^33^	*U*^12^	*U*^13^	*U*^23^
F1	0.0904 (10)	0.0706 (9)	0.1334 (15)	−0.0264 (8)	0.0185 (10)	0.0103 (9)
O1	0.0994 (11)	0.0750 (10)	0.0495 (8)	−0.0053 (8)	0.0428 (8)	−0.0079 (7)
N1	0.0596 (9)	0.0653 (10)	0.0411 (8)	−0.0057 (7)	0.0275 (7)	−0.0050 (7)
N2	0.0694 (11)	0.0849 (14)	0.0739 (13)	0.0008 (10)	0.0208 (10)	−0.0273 (10)
N3	0.0781 (12)	0.0777 (12)	0.0517 (11)	0.0052 (9)	0.0310 (10)	−0.0057 (8)
N4	0.1118 (19)	0.160 (3)	0.0615 (14)	0.0438 (19)	0.0055 (14)	−0.0281 (15)
C1	0.0507 (10)	0.0542 (10)	0.0484 (9)	0.0043 (8)	0.0247 (8)	−0.0003 (8)
C2	0.0684 (12)	0.0663 (13)	0.0534 (11)	0.0030 (10)	0.0276 (9)	0.0079 (9)
C3	0.0763 (14)	0.0614 (13)	0.0873 (16)	0.0025 (11)	0.0396 (13)	0.0198 (11)
C4	0.0589 (12)	0.0535 (12)	0.0897 (17)	−0.0022 (9)	0.0186 (11)	0.0052 (11)
C5	0.0646 (12)	0.0637 (13)	0.0652 (13)	−0.0030 (10)	0.0146 (10)	−0.0036 (10)
C6	0.0621 (11)	0.0621 (12)	0.0484 (10)	−0.0026 (9)	0.0231 (9)	−0.0008 (8)
C7	0.0568 (10)	0.0593 (11)	0.0429 (9)	0.0079 (8)	0.0234 (8)	−0.0033 (8)
C8	0.0651 (12)	0.0739 (13)	0.0620 (12)	−0.0080 (10)	0.0292 (10)	−0.0187 (10)
F2	0.1302 (15)	0.1254 (16)	0.174 (2)	−0.0711 (13)	0.0557 (15)	−0.0489 (15)
O2	0.1083 (13)	0.0784 (10)	0.0623 (9)	−0.0201 (9)	0.0561 (9)	−0.0135 (7)
N5	0.0633 (10)	0.0632 (10)	0.0437 (8)	−0.0014 (7)	0.0276 (7)	0.0018 (7)
N6	0.1168 (17)	0.1021 (17)	0.0610 (12)	−0.0385 (14)	0.0453 (12)	−0.0225 (11)
N7	0.0765 (12)	0.0788 (12)	0.0501 (9)	−0.0018 (11)	0.0292 (9)	−0.0103 (9)
N8	0.0906 (15)	0.123 (2)	0.0712 (13)	−0.0231 (15)	0.0300 (12)	−0.0307 (13)
C9	0.0530 (10)	0.0571 (11)	0.0538 (10)	0.0049 (8)	0.0239 (8)	−0.0017 (8)
C10	0.0691 (13)	0.0655 (13)	0.0654 (13)	−0.0018 (10)	0.0334 (10)	0.0007 (10)
C11	0.0871 (17)	0.0708 (15)	0.106 (2)	−0.0101 (13)	0.0538 (16)	−0.0003 (14)
C12	0.0779 (16)	0.0806 (18)	0.112 (2)	−0.0217 (13)	0.0351 (16)	−0.0251 (16)
C13	0.0863 (17)	0.096 (2)	0.0774 (17)	−0.0134 (15)	0.0181 (14)	−0.0200 (15)
C14	0.0750 (14)	0.0762 (15)	0.0578 (12)	−0.0036 (11)	0.0201 (10)	−0.0046 (11)
C15	0.0615 (11)	0.0580 (11)	0.0472 (10)	0.0029 (8)	0.0262 (9)	0.0004 (8)
C16	0.0717 (13)	0.0722 (13)	0.0502 (11)	−0.0077 (10)	0.0270 (9)	−0.0008 (9)

### Geometric parameters (Å, º)

**Table:**

F1—C4	1.353 (3)	F2—C12	1.358 (3)
O1—C7	1.224 (2)	O2—C15	1.225 (2)
N1—C7	1.343 (2)	N5—C15	1.341 (2)
N1—C1	1.409 (2)	N5—C9	1.411 (3)
N1—H1	0.8600	N5—H5A	0.8600
N2—N3	1.220 (3)	N6—N7	1.202 (3)
N2—C8	1.455 (3)	N6—C16	1.465 (3)
N3—N4	1.105 (3)	N7—N8	1.120 (3)
C1—C2	1.389 (3)	C9—C10	1.385 (3)
C1—C6	1.390 (3)	C9—C14	1.391 (3)
C2—C3	1.386 (3)	C10—C11	1.382 (3)
C2—H2	0.9300	C10—H10	0.9300
C3—C4	1.368 (4)	C11—C12	1.374 (4)
C3—H3	0.9300	C11—H11	0.9300
C4—C5	1.359 (3)	C12—C13	1.358 (4)
C5—C6	1.374 (3)	C13—C14	1.368 (4)
C5—H5	0.9300	C13—H13	0.9300
C6—H6	0.9300	C14—H14	0.9300
C7—C8	1.511 (3)	C15—C16	1.508 (3)
C8—H8A	0.9700	C16—H16A	0.9700
C8—H8B	0.9700	C16—H16B	0.9700
			
C7—N1—C1	127.90 (16)	C15—N5—C9	127.53 (16)
C7—N1—H1	116.0	C15—N5—H5A	116.2
C1—N1—H1	116.0	C9—N5—H5A	116.2
N3—N2—C8	116.02 (19)	N7—N6—C16	115.87 (19)
N4—N3—N2	171.2 (3)	N8—N7—N6	171.2 (2)
C2—C1—C6	119.27 (18)	C10—C9—C14	119.6 (2)
C2—C1—N1	123.51 (17)	C10—C9—N5	123.08 (18)
C6—C1—N1	117.20 (17)	C14—C9—N5	117.35 (18)
C3—C2—C1	119.3 (2)	C11—C10—C9	119.4 (2)
C3—C2—H2	120.3	C11—C10—H10	120.3
C1—C2—H2	120.3	C9—C10—H10	120.3
C4—C3—C2	119.4 (2)	C12—C11—C10	119.1 (3)
C4—C3—H3	120.3	C12—C11—H11	120.4
C2—C3—H3	120.3	C10—C11—H11	120.4
F1—C4—C5	118.5 (2)	C13—C12—F2	119.4 (3)
F1—C4—C3	119.0 (2)	C13—C12—C11	122.5 (2)
C5—C4—C3	122.4 (2)	F2—C12—C11	118.2 (3)
C4—C5—C6	118.4 (2)	C12—C13—C14	118.5 (3)
C4—C5—H5	120.8	C12—C13—H13	120.7
C6—C5—H5	120.8	C14—C13—H13	120.7
C5—C6—C1	121.11 (19)	C13—C14—C9	120.9 (2)
C5—C6—H6	119.4	C13—C14—H14	119.6
C1—C6—H6	119.4	C9—C14—H14	119.6
O1—C7—N1	124.23 (19)	O2—C15—N5	124.02 (19)
O1—C7—C8	122.15 (17)	O2—C15—C16	121.86 (18)
N1—C7—C8	113.61 (16)	N5—C15—C16	114.10 (16)
N2—C8—C7	110.25 (17)	N6—C16—C15	107.63 (17)
N2—C8—H8A	109.6	N6—C16—H16A	110.2
C7—C8—H8A	109.6	C15—C16—H16A	110.2
N2—C8—H8B	109.6	N6—C16—H16B	110.2
C7—C8—H8B	109.6	C15—C16—H16B	110.2
H8A—C8—H8B	108.1	H16A—C16—H16B	108.5
			
C7—N1—C1—C2	22.8 (3)	C15—N5—C9—C10	23.2 (3)
C7—N1—C1—C6	−158.76 (19)	C15—N5—C9—C14	−158.3 (2)
C6—C1—C2—C3	1.5 (3)	C14—C9—C10—C11	1.4 (3)
N1—C1—C2—C3	179.95 (18)	N5—C9—C10—C11	179.8 (2)
C1—C2—C3—C4	−0.4 (3)	C9—C10—C11—C12	−0.3 (4)
C2—C3—C4—F1	179.9 (2)	C10—C11—C12—C13	−1.2 (5)
C2—C3—C4—C5	−1.3 (4)	C10—C11—C12—F2	180.0 (2)
F1—C4—C5—C6	−179.38 (19)	F2—C12—C13—C14	−179.6 (3)
C3—C4—C5—C6	1.8 (4)	C11—C12—C13—C14	1.6 (5)
C4—C5—C6—C1	−0.6 (3)	C12—C13—C14—C9	−0.5 (4)
C2—C1—C6—C5	−1.0 (3)	C10—C9—C14—C13	−1.0 (4)
N1—C1—C6—C5	−179.57 (18)	N5—C9—C14—C13	−179.5 (2)
C1—N1—C7—O1	−0.3 (3)	C9—N5—C15—O2	−0.3 (3)
C1—N1—C7—C8	−178.95 (18)	C9—N5—C15—C16	178.13 (18)
N3—N2—C8—C7	−106.1 (2)	N7—N6—C16—C15	−175.4 (2)
O1—C7—C8—N2	24.7 (3)	O2—C15—C16—N6	−13.6 (3)
N1—C7—C8—N2	−156.63 (18)	N5—C15—C16—N6	168.00 (19)

### Hydrogen-bond geometry (Å, º)

*Cg*1 and *Cg*2 are the centroids of the C1–C6 and C9–C14 benzene rings, respectively.

**Table:**

*D*—H···*A*	*D*—H	H···*A*	*D*···*A*	*D*—H···*A*
N1—H1···O1^i^	0.86	2.17	2.921 (2)	146
N5—H5*A*···O2^i^	0.86	2.13	2.885 (2)	146
C4—F1···*Cg*1^ii^	1.35 (1)	3.76 (1)	3.563 (2)	72 (1)
C12—F2···*Cg*2^iii^	1.36 (1)	3.98 (1)	3.942 (2)	79 (1)

Symmetry codes: (i) *x*, −*y*+1/2, *z*+1/2; (ii) −*x*+1, −*y*+1, −*z*+1; (iii) −*x*+2, −*y*+1, −*z*+2.
